# A unique type of fully covered metal stent for the management of post liver transplant biliary anastomotic strictures

**DOI:** 10.1186/s12876-020-01479-6

**Published:** 2020-10-07

**Authors:** Ben Warner, Phillip Harrison, Muhammad Farman, John Devlin, David Reffitt, Yasser El-Sherif, Shirin E. Khorsandi, Andreas Prachalias, Miriam Cortes Cerisuelo, Krish Menon, Wayel Jassem, Parthi Srinivasan, Hector Vilca-Melendez, Michael Heneghan, Nigel Heaton, Deepak Joshi

**Affiliations:** Institute of Liver Studies, King’s College Hospital NHS Foundation Trust, Denmark Hill, London, SE5 9RS UK

**Keywords:** ERCP, Anastomotic strictures, FCSEMS, Biliary

## Abstract

**Background:**

We report our experience of treating anastomotic strictures using a novel type of fully covered metal stent (FCSEMS). This stent, known as the Kaffes Stent, is short-length with an antimigration waist and is easily removable due to long retrieval wires deployed within the duodenum.

**Methods:**

Sixty-two patients underwent ERCP and Kaffes stent insertion for post-transplant anastomotic strictures following confirmation of a stricture on MRCP. These patients were retrospectively analysed for immediate and long-term stricture resolution, improvement in symptoms and liver function tests (LFTs), stricture recurrence and complication rates.

**Results:**

Of the 56 patients who had their stent removed at the time of analysis, 54 (96%) had immediate stricture resolution and 42 continued to have long-term resolution (mean follow-up period was 548 days). Of the 16 patients with symptoms of biliary obstruction, 13 had resolution of their symptoms. Overall, there was a significant improvement in LFTs after stent removal compared to before stent insertion. Complication rates were 15% with only one patient requiring biliary reconstruction.

**Conclusions:**

The Kaffes stent is effective and safe at resolving post liver transplant biliary anastomotic strictures.

## Background

Anastomotic strictures are isolated, short-length strictures affecting 4–9% of patients post-liver transplantation, typically associated with technical factors such as bile leaks, the length of the donor bile duct, suture placement and size discrepancy [1, 2]. They typically develop 5–8 months after transplantation [3].

Stenting has historically been with plastic stents, on a 3-monthly basis for up to a year. Although multiple plastic stent (MPS) placement has higher efficacy at resolving strictures compared to single plastic stents (SPS), plastic stents in general are at risk of stent occlusion, and often require multiple ERCPs before stricture resolution is achieved [4]. FCSEMS (fully covered self expanding metallic stents) have been shown to resolve benign strictures; however, stent migration can occur because the centre of the stent does not always overlie the stricture [5–7].

Kaffes stents (Taewoong Medical) are a novel type of FCSEMS that have a short-length, an antimigration waist, and long removal wires which lie within the duodenum for easy removal. The ends of the stents are larger in diameter than the mid-point. This unique design reduces stent migration by producing a radial force against the stricture, contributing to better outcomes. Previous randomised trials have illustrated their success with resolving anastomotic strictures when compared to plastic stenting [8].

## Methods

Data on patients in whom Kaffes stents were inserted between December 2016 and February 2020 were retrospectively analysed for stricture resolution, adverse event rates and improvements in both symptoms and liver function tests (LFTs). Ethics approval was not required as inserting this type of stent was already introduced as standard of care at our institution.

### Inclusion criteria

This was a single centre study at King’s College Hospital, London. All patients included had duct-to-duct biliary anastomoses and were referred for ERCP based on a radiologically confirmed anastomotic stricture with proximal biliary dilatation on MRCP (magnetic resonance cholangiopancreatography). MRCPs were performed either following deterioration of LFTs, new cholestatic symptoms or in the asymptomatic patients if their routine follow-up ultrasounds showed biliary dilatation. Patients with non-anastomotic complex hilar strictures were excluded.

### Method for insertion

**Fig. 1:**
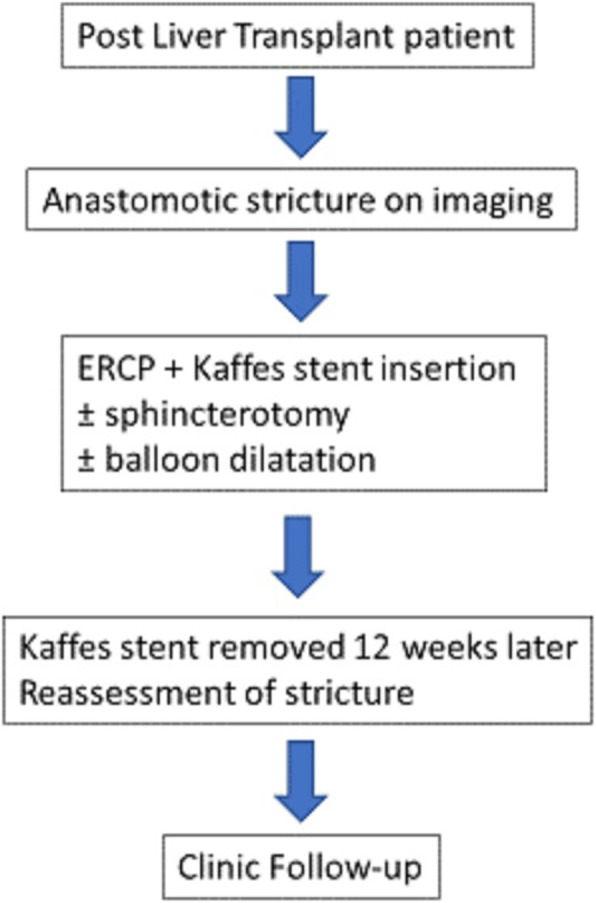
Protocol for Kaffes stent insertion. ERCP: Endoscopic retrograde cholangiopancreatography

Two types of Kaffes stent were inserted – a 40 mm by 10 mm stent and a 40 mm by 8 mm stent. All Kaffes stents were inserted over a HydraJAG guidewire, 0.035-in. (Boston Scientific). Stents aimed to be removed 12 weeks after insertion with stent forceps and a cholangiogram was performed to assess whether the stricture had resolved or whether further stenting was required. Decisions to perform a sphincterotomy or balloon dilatation were made by the endoscopist at the time of the procedure but followed no pre-determined protocol (Fig. 1).

### Definitions

Initial resolution of a stricture was defined as either where there was no stricture demonstrated on the cholangiogram (at the time of the second ERCP), or if there was still a stricture, whether a 12 mm extraction balloon was easily able to pass through. Recurrence was defined as where the stricture occurred within the follow-up period. Long-term stricture resolution was defined by the absence of recurrence within the follow-up period. Complications were found from discharge summaries. LFTs were analysed before stent insertion and after removal in patients with resolved strictures only.

### Statistical analysis

Categorical and continuous variables were analysed using the Fisher’s exact test and independent samples t-test respectively. A paired sample t-test was used to assess for improvement in LFTs. 2 × 2 contingency tables assessed significant differences between categorical variables www.graphpad.com/quickcalcs/contingency1/.

## Results

Over the period investigated, 761 patients were transplanted. Sixty-two of these patients had a Kaffes stent inserted for post-transplant anastomotic strictures, mean age of 53 (SD 11.9, range 13–72) years; 1 patient had a living-related donor right lobe graft; of the remainder 68% were DBD (donation after brain death) grafts. The mean CIT (cold ischaemic time) was 8.4 h (±2.4) for the DBD group and 8.9 h (±2.5) for the DCD (donation after circulatory death) group (Table 1). The aetiologies for liver disease are shown in Table 2. 13 (21%) patients had previous plastic stenting and 1 patient had had the traditional type of longer-length FCSEMS (Wallflex™, Boston Scientific) without stricture resolution. Two patients had had previous stenting for post anastomotic biliary leaks. The median time between the transplant and Kaffes insertion was 10 months (IQR 34). 16 (26%) patients required stenting for stricturing within 3 months of transplantation. Thirty-eight patients had balloon dilatations of strictures prior to stent insertion. Twenty-nine had a sphincterotomy at the time of stent insertion, whilst another patient had had a previous sphincterotomy.

**Table 1:** Highlights the main outcomes and specific factors of the Kaffes stent.

No. of patients inserted	62
No. of patients removed	56
Immediate stricture resolution	54/56 (96%)
Long-term stricture resolution (%)	42/52^a^ (81%)
Complications (%)	9/62 (15%)
Biliary reconstruction	1
Mean age (years)	53
Females (%)	26 (42%)
Symptomatic (%)	16 (26%)
DBD (%)	36/53 (68%)
CIT (Hours) (±SD)	8.6 (2.4)

*DBD* Donation after brain death; *CIT* Cold ischaemic time. ^a^ One patient died of frailty post-transplant 13 months after a Kaffes stent successfully resolved the stricture and another was re-transplanted for chronic rejection so for the purpose of this study, both were not included in the analysis for long-term stricture resolution

**Table 2:** The aetiologies of liver disease for patients stented.

Aetiology	No. of Patients	Aetiology	No. of Patients
Alcoholic liver disease	14	Drug-induced liver failure	1
Hepatitis C	4	Primary Hyperoxaluria	2
Primary biliary cirrhosis	4	Cystic Fibrosis related cirrhosis	1
Budd Chiari	3	NASH	4
Autoimmune hepatitis	3	Polycystic	2
Hepatitis B	2	PFIC	1
HCC	12	PSC	1
Acute liver failure, cause unknown	4	Paracetamol toxicity	3
Wilson’s disease	1		

*HCC* Hepatocellular Carcinoma; Non-alcoholic Steatohepatitis; *PFIC* Primary Familial Intrahepatic Cholestasis

### Immediate stricture resolution (Fig. 2)

To date, 56 patients have had their Kaffes stent removed, of whom 54 (96%) had immediate stricture resolution at the time of stent removal. There was no relationship between stent size, balloon dilatations (*N* = 33, *P* = 1.00) and/or sphincterotomies (*N* = 26, *P* = 1.00) and stricture resolution. Of the 2 patients in whom the Kaffes stent had failed to resolve stricturing, 1 went onto have stricture resolution with a plastic stent insertion, whilst the other had a traditional longer-type of FCSEMS inserted without successful stricture resolution.

**Fig. 2:**
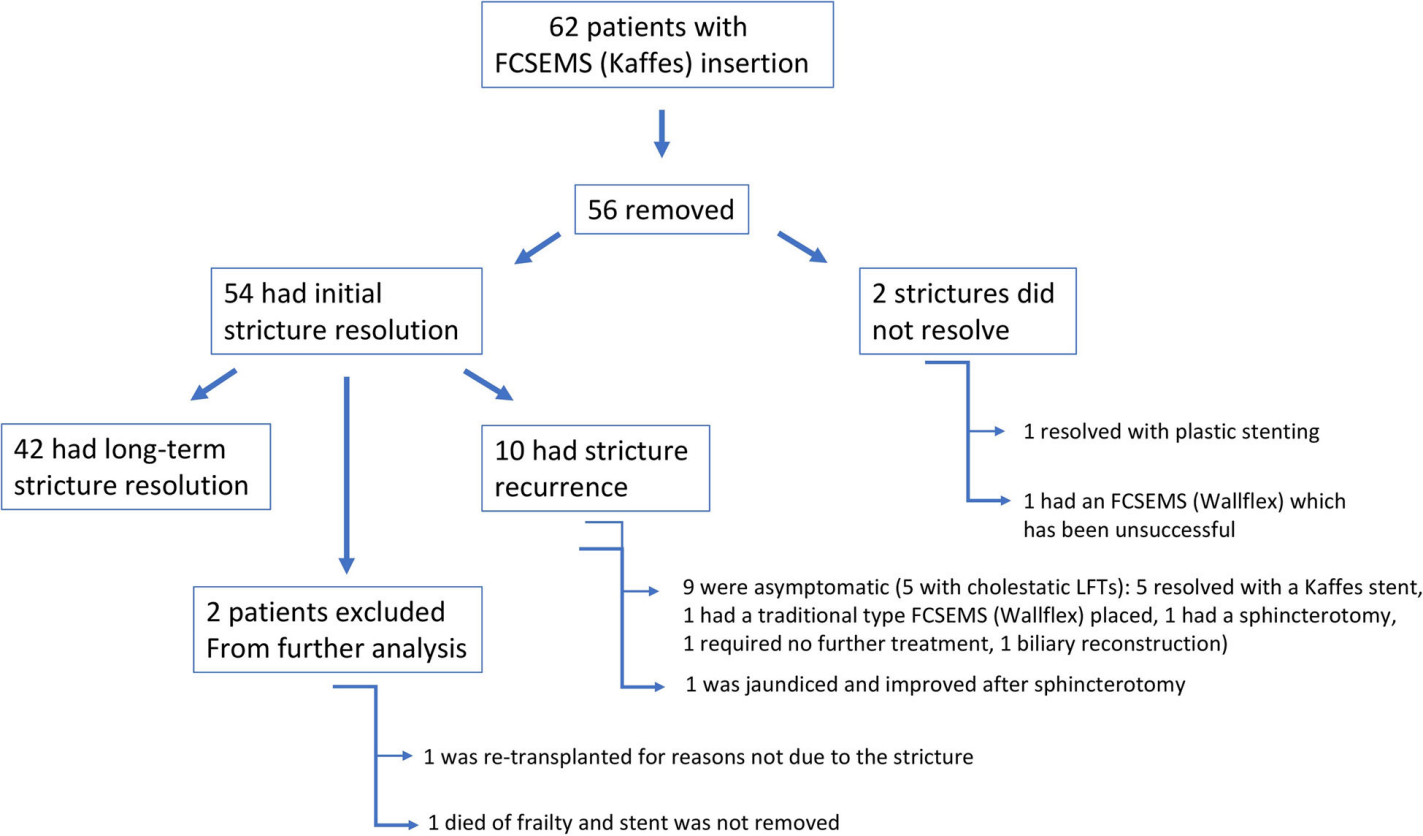
A flow chart of outcomes in the FCSEMS group

Of the 54 patients in whom there was immediate stricture resolution, 1 died of frailty a year after transplant and another was re-transplanted due to chronic rejection, both having no relationship to their initial anastomotic stricture; these patients were excluded from the overall analysis.

### Long-term stricture resolution

Overall, 42/52 (81%) patients went onto have long-term stricture resolution with no recurrence (mean follow-up period was 548 days (SD 256, range 13–1097 days). Of the 10 patients who had recurrence (mean time to recurrence was 224 days (SD 200, range 37–575 days), 1 patient became jaundiced 3 months after stent removal, but instead of further stenting, a sphincterotomy was enough to improve drainage. Nine had asymptomatic recurrence on imaging (5 with cholestatic LFTs), of whom 5 had a further Kaffes placed, 1 had the traditional FCSEMS placement, another improved with sphincterotomy. Only 1 patient required biliary reconstruction as the wire failed to pass through the stricture at their ERCP.

### Improvement in symptoms

Forty-six patients were asymptomatic with an anastomotic stricture on imaging (37 with cholestatic LFTs). Sixteen patients were symptomatic (6 jaundiced, 4 pruritic, 6 cholangitic). Of these, symptoms resolved in 13 (3 clinically jaundiced, 4 pruritic, 6 cholangitic). Of the 3 patients who continued to be jaundiced after stent insertion, 2 had their stent removed within 4 weeks of insertion, the 3rd after 86 days; all 3 were found to have stricture resolution. Two of these patients were found to have papillary stenosis and improved after sphincterotomy; the other patient required re-transplantation due to chronic rejection. Overall, there was a significant improvement in LFTs when comparing LFTs before stent insertion and after removal in those 54 patients for whom their stricture had resolved (see Table 3).

**Table 3:** The mean LFTs in patients before stent insertion compared to after removal in patients for whom their stricture had resolved (*n* = 54).

LFTs	Mean before stent (SD)	Mean after stent (SD)	*P* value	Upper Limit of normal	Number of abnormal results before stent	Number of abnormal results after stent
GGT (IU/L)	513 (538)	193 (320)	< 0.01	72 (IU/L)	44	32
AST (IU/L)	101 (179)	41.4 (32.1)	0.03	22 (IU/L)	22	13
ALP (IU/L)	313 (322)	169 (109)	< 0.01	129 (IU/L)	39	31
Bili (μmol/L)	26.7 (46.1)	14.0 (13.6)	0.03	21 (μmol/L)	19	8

The table also displays the number of abnormal results before and after stent insertion and the laboratory’s upper limit of normal. *LFTs* Liver function tests; *SD* Standard deviation

### Complications

Overall, 9 (15%) of the 62 patients had a complication: 3 patients developed pancreatitis after the Kaffes stent insertion, 4 developed cholangitis, 1 after insertion and 3 following stent removal, 1 patient had a wire-guided perforation of the bile duct without complication, 1 had the retrieval wires uncoil. There was no associated mortality.

Of the stents that were removed at the time of writing, all were removed successfully (mean of 114 days (SD 70), range 3–345 days), although as above, 1 stent needed 2 attempts because the removal wires uncoiled. Stent removal for a patient 345 days after insertion was delayed due to pregnancy; the stent was removed easily without complications.

## Discussion

**Fig. 3:**
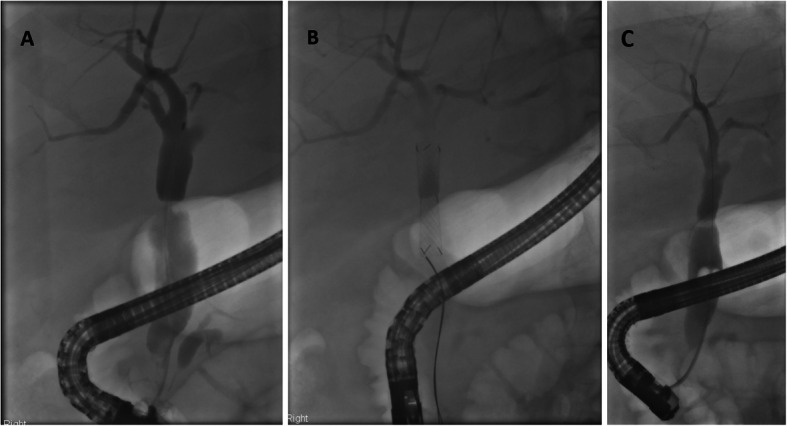
The cholangiograms are of a patient who underwent liver transplantation in 2014 for HCC with underling NASH cirrhosis (DBD graft, CIT 8 h). 31 months later they developed cholestatic liver enzymes and imaging showed an anastomotic stricture. They underwent an ERCP and plastic stent insertion but the stent migrated. At the 2nd ERCP (Fig. 3**a**), the cholangiogram demonstrated the persistence of this stricture. The stricture was dilated using an 8 mm Hurricane Balloon (Boston Scientific) and an 8x40mm Kaffes Stent inserted (Fig. 3**b**). 3 radiopaque markers on either side of the stent confirm the stent’s position – the top markers represent where the stent begins to be deployed and the middle markers where it should sit over the stricture. Biochemistry immediately improved. The stent was removed 63 days later with complete resolution of the stricture as shown (Fig. 3**c**)

**Fig. 4:**
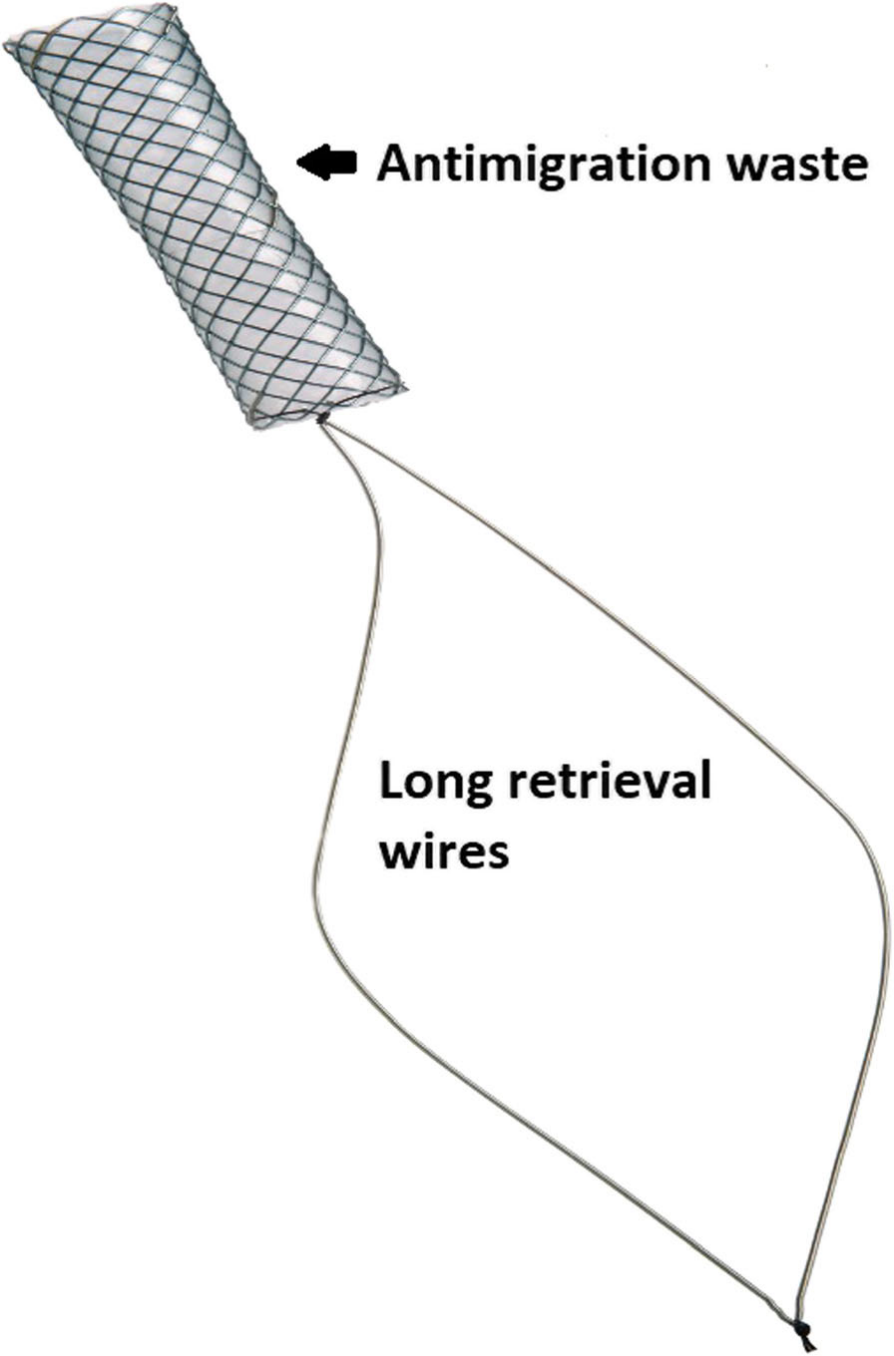
Shows the unique design of the Kaffes Stent with its short-stent length, antimigration waste and long retrieval wires

Our results confirm that the Kaffes stent is effective at resolving anastomotic strictures with only one patient requiring biliary reconstruction. Figure 3 highlights an example of a patient whose anastomotic stricture was successfully resolved with the Kaffes Stent. This effectiveness appears to be due to its unique design, where its short-length and variable diameter places a radial force outwards so as to resolve the stricture whilst also preventing stent migration (Fig. 4).

Our experience, however, has highlighted a ‘learning curve’ with using the Kaffes stent. One issue is the delivery of the deployment wires into the duodenum. We recommend the use of forceps, inserted through the scope, to pull the wires out. Care should be taken not to just withdraw the scope with the wires inside it, as this could dislodge the stent. Another issue is that the stents can collect sludge and debris, and this is evident at the time of stent removal. This finding was associated with 3 of our patients developing cholangitis after removal; a balloon trawl is advised to remove any remaining debris after the stent has been removed. One patient became pregnant after her Kaffes stent was inserted. It was therefore not removed for over a year but was successfully removed without complication. However, the longer the stents are left in for, the more likely they are to collect sludge and debris. The use of prophylactic ursodeoxcholic acid may be of some benefit although we have not adopted this approach at the time of writing. Twelve weeks is therefore the recommended maximum length of time they should be left for. Finally, the Kaffes stent should not be deployed for anastomotic strictures too close to the hilum (< 2 cm) as they could result in biliary obstruction.

There was no relationship between the patients who had sphincterotomies or balloon dilatations, and successful resolution of the anastomotic stricture using the Kaffes stent. The stent is deployed from an 8.5 Fr delivery system so unless the stricture is very tight, the stents were mostly able to cross the stricture without dilatation.

A number of studies have compared different types of stent for the resolution of anastomotic strictures. Cantù et al. performed a large prospective study comparing 3 groups: single plastic stenting (SPS), multiple plastic stenting (MPS) and the traditional longer-length FCSEMS [4]. SPS placement performed the least well with a stricture resolution rate of 61%. Both MPS and FCSEMS placement were associated with a higher rate of stricture resolution (88%), although there was a 19% migration rate within the FCSEMS group.

A meta-analysis by Lee et al. compared the efficacy of MPS versus FCSEMS [9]. The stricture resolution rate was 84.5% (ranging from 63 to 100%) with MPS compared to a significantly lower stricture resolution rate of 75% (53 to 81%) with FCSEMS. Recurrence rates were no different between the groups but complication rates, mainly migration (accounting for between 4 and 46%), were higher in the FCSEMS group. The number of procedures performed to achieve stricture resolution was not compared.

Visconti et al. performed a meta-analysis of 4 randomized control trials totalling 205 patients, specifically comparing the number of procedures and overall costs [10]. It had previously been assumed that the cost of FCSEMS placement was higher due to the price of the stents. No difference was observed between stricture resolution rates, recurrence, and adverse events between MPS and FCSEMS. However, there were significantly fewer ERCPs in the FCSEMS group and hence the cost was lower overall (average $8288.5 versus $18,580 in the FCSEMS versus MPS group respectively).

The benefits of plastic stents are that they are easily inserted, inexpensive and easily replaceable. MPS have been shown to result in high resolution rates. However, the disadvantages are that they have an increased risk of stent occlusion because of their relatively small diameters and often require multiple sessions (median of 3 to 5 procedures) which have the potential to increase complications such as pancreatitis, cholangitis and perforation [9]. The efficacy of traditional longer-length FCSEMS in meta-analyses is similar to MPS, but with fewer ERCPs required, and have the potential to be cheaper overall. They do, however, have the downside of being associated with stent migration.

In our study, the Kaffes stent resulted in long-term stricture resolution of 81% after just one placement and owing to its unique design, no stent migration occurred. This offers a clear benefit over not only the traditional longer-length FCSEMS but also over MPS, as fewer procedures may mean a greater acceptance by patients and fewer overall complications.

Although a small comparison study between the Kaffes stent and MPS confirmed the Kaffes stent to be more effective, larger prospective multicentre studies are still needed [8].

## Conclusion

In conclusion, our preliminary observations indicate that the Kaffes stent is highly effective at treating anastomotic strictures. Complication rates are low and stent migration is not a feature. Patients require fewer ERCPs than with MPS. These findings will improve the quality of life for post-transplant patients avoiding repeated ERCPs and potentially biliary reconstruction. Further larger multicentre studies are required to understand the efficacy of this stent in the management of anastomotic biliary strictures post liver transplantation.

## Data Availability

The datasets used and/or analysed during the current study are available from the corresponding author on reasonable request.

## Abbreviations

FCSEMS: Fully covered metal stent
LFTs: Liver function tests
MR: Magnetic resonance
CIT: Cold ischaemic time
DBD: Donation after brain death
DCD: Donation after circulatory death
MPS: Multiple plastic stenting
